# Antimicrobial Effects of Platelet-Rich Plasma and Platelet-Rich Fibrin: A Scoping Review

**DOI:** 10.7759/cureus.51360

**Published:** 2023-12-30

**Authors:** Karan CL, Madhan Jeyaraman, Naveen Jeyaraman, Swaminathan Ramasubramanian, Manish Khanna, Sankalp Yadav

**Affiliations:** 1 Orthopaedics, Sanjay Gandhi Institute of Trauma & Orthopaedics, Bengaluru, IND; 2 Orthopaedics, ACS Medical College and Hospital, Dr. MGR Educational and Research Institute, Chennai, IND; 3 Orthopaedics, Government Medical College, Omandurar Government Estate, Chennai, IND; 4 Orthopaedics, Autonomous State Medical College, Ayodhya, IND; 5 Internal Medicine, Shri Madan Lal Khurana Chest Clinic, New Delhi, IND

**Keywords:** antibacterial, centrifugation, platelet-rich fibrin, platelet-rich plasma (prp), ­wound healing

## Abstract

Platelet-rich plasma (PRP), derived from the centrifugation and subsequent separation of whole blood, results in an unusually high concentration of platelets. A newer form of platelet concentrate, platelet-rich fibrin (PRF), has also been developed. There has been significant research into the therapeutic effects of PRP, particularly in enhancing wound healing and preventing infections in surgical wounds. This scoping review aims to thoroughly evaluate preclinical and clinical evidence regarding the antimicrobial effects of PRP and PRF. In conducting this review, 612 records were examined, and 12 articles were selected for inclusion. The studies reviewed include preclinical research, such as in-vitro and in-vivo studies, and clinical trials involving human participants. The current clinical evidence suggests a notable trend towards the antimicrobial capabilities of PRP and PRF, underscoring their potential benefits in treating wounds. The application of PRP and PRF in wound management shows encouraging outcomes, but further investigation is needed to optimize their use as antimicrobial agents. Additional research, particularly randomized controlled trials, is essential to substantiate their antimicrobial effectiveness in specific diseases and types of wounds, considering their potential impact on clinical results.

## Introduction and background

Platelet concentrates are obtained from the patient's blood. In present times, the use of autologous platelet concentrates (PCs) has gained popularity in multiple medical fields, including oral surgery and dentistry, as well as the healing of soft tissues such as ligaments, tendons, and muscles in orthopedics. Furthermore, this method has also found application in dermatology, cosmetic surgery, and plastic surgery [1,2].

Platelet-rich plasma (PRP) has emerged as a novel approach in the field of healing and tissue regeneration. PRP contains a platelet concentration that is significantly higher, approximately three to five times, than what is typically found in whole blood. This increased concentration of platelets enhances the clotting mechanism, offering potential benefits in wound healing. The regenerative properties of PRP are largely attributed to the release of various growth factors. These include platelet-derived growth factor, epithelial growth factor (EGF), vascular endothelial growth factor, transforming growth factors (TGF-β1 and TGF-β2), and insulin-like growth factor (IGF), which collectively contribute to its therapeutic effects in tissue repair and regeneration [3,4]. The wound healing capacity of PRP can, therefore, be related to the amounts of GFs in the wound site following platelet degranulation [5]. PRP, in addition, harbors immunological mediators, enzymes as well as their suppressors, and plasma complement, entities that have been associated with the regulation of bacterial populations, the restoration of tissue integrity, as well as the recuperation of wounds [6-9]. The leukocyte concentration increases five to tenfold in many PRP preparations after PRP processing. There are conflicting reports that suggest that higher leukocyte concentrations could influence the inflammatory response by recruiting immune cells and stabilizing the matrix [10,11]. Other reports suggest that higher leukocytes could exacerbate the inflammatory response by secreting pro-inflammatory proteases, delaying healing [12].

Platelet-rich fibrin (PRF) represents a more recent iteration of platelet concentrate, which facilitates the process of healing through the integration of bone and soft tissue [13]. Leukocyte and platelet-rich fibrin (L-PRF) are obtained from individuals' blood through centrifugation at a relative centrifugal field of 700 for 12 minutes without any additional substances [14]. The Platelet-Rich Fibrin (PRF) is a tridimensional structure composed of fibrin, housing numerous self-originating cellular elements, including platelets, neutrophils, and macrophages [15]. The resulting fibrin matrix facilitates the gradual release of growth factors and assumes the role of a storage scaffold [16]. PRF has exhibited substantial potential as a therapeutic modality in the field of dentistry, catering to the regrowth needs of both soft and hard dental tissues [17]. Recent studies have demonstrated that compared to fixed-angle centrifugation, horizontal centrifugation of PRF improved cell layer separation and resulted in a fourfold increase in immune cell counts [18]. The antimicrobial effects of PRF obtained through horizontal centrifugation (H-PRF) may be more pronounced in comparison to those of PRF or L-PRF.

The wound-healing process is mediated by molecular interactions involving mesenchymal cell recruitment, proliferation, and extracellular matrix regeneration. Inflammation, coagulation, epithelialization, granulation tissue formation, and tissue remodeling are all part of the healing process [19]. Platelet aggregation at the location of tissue damage generates a fibrin clot that facilitates the process of hemostasis and attracts an assortment of cell populations to the site. Cytokines and growth factors govern these cellular activities [20]. These platelets undergo degranulation, thereby liberating histamine, serotonin, and bioactive factors. This action attracts inflammatory cells, such as neutrophils and macrophages, to the vicinity. Reactive oxygen species (ROS) are produced during the process of wound repair, leading to peroxidation of the lipid constituents of the cellular membrane and alterations in various antioxidant enzyme systems [21]. The disparity between an abundance of oxidative stress and the presence of antioxidative systems is responsible for the occurrence of cellular senescence [22].

In recent years, the use of PRP for its antimicrobial properties, along with its wound-healing properties, has been widely explored. Postoperative acute infections [23,24], chronic wound infections [25-27], or osteomyelitis [28-31] may be prevented or treated with this property of PRP. PRP's antimicrobial properties could be attributed to platelet immunological characteristics. During wound healing, leukocytes recruited to infection sites trigger signaling pathways that phagocytose and kill microorganisms [8]. Studies have shown that after activation, platelets can release kinocidins (such as CXCL4, CXCL7, and CXCL5) that have antibacterial and antifungal properties [32-34]. Platelet lysate (PL) has recently been studied for its efficacy against various bacteria [35]. Reactive oxygen species (ROS) generated by platelets are capable of adhering to, phagocytosing, and taking part in antibody-dependent cellular cytotoxicity [36].

The following research questions were addressed by this scoping review: a) Do PRP and PRF have antibacterial properties?; b) What is the mechanism of antibacterial action of PRP? c) Does PRP exert bactericidal or bacteriostatic effects? d) Does the use of various agents provide a synergistic effect? By addressing these inquiries, the fundamental aim of this comprehensive examination was to scrutinize the mechanisms elucidating the antimicrobial efficacy of platelet preparations in light of the accessible preclinical and clinical grounds.

## Review

Methods

The literature review adhered to specific inclusion and exclusion criteria. Search terms included "Platelet-rich plasma," "platelet-rich fibrin," "PRP," "PRF," "plasma concentrate," "antimicrobial," and "bacterial infections." Relevant studies from 2000 to 2023 were sourced from PubMed, MEDLINE, Scopus, Web of Science, and Embase databases to explore peer-reviewed medical literature. The quality and relevance of studies were evaluated, and those meeting the predefined criteria were included in the review. Inclusion was not contingent on whether studies reported positive or negative outcomes related to PRP and PRF.

The scope of this review covers both experimental and quasi-experimental research, including randomized controlled trials, non-randomized controlled trials, pre- and post-studies, and interrupted time-series studies. It also involves analytical observational studies such as case-control, prospective and retrospective cohort studies, and analytical cross-sectional studies. Descriptive observational studies, including case series, individual case reports, and descriptive cross-sectional studies, are also considered for potential inclusion. Systematic reviews meeting the criteria are evaluated depending on the research question.

This review includes pre-clinical (both in vitro and in vivo) and clinical research, focusing on the antibacterial properties of platelet-rich plasma and platelet-rich fibrin. In vitro studies conducted in controlled environments outside living organisms and in vivo studies undertaken within living organisms are both considered. Clinical studies involving human subjects are also a crucial component of this research.

The studies considered were evaluated for their inclusion and methodological quality. For each study, the publication, type of investigation and size of the sample, the purpose of the investigation and measurement of the result, PRP/PRF utilized, types of bacteria targeted, activator employed, and transformations in result measurements are declared. The investigations not included in the process of extracting data were those composed in a foreign language, where the entire text was not accessible, not subject to peer review, case series/reports, and in cases where data relevant to PRP/PRF could not be derived.

**Figure 1:**
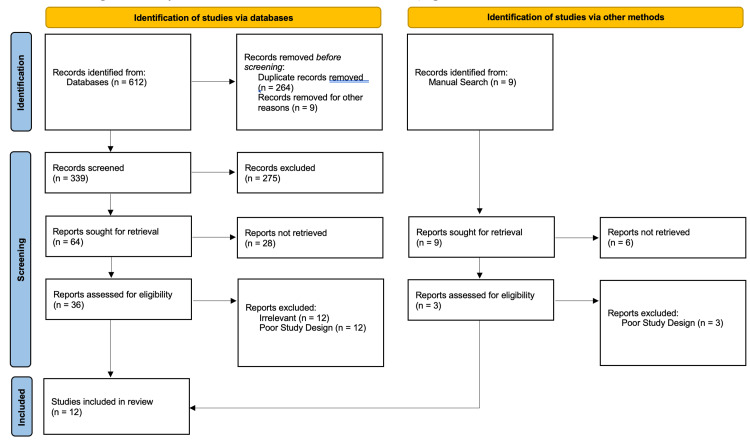
Selection of literature included in the scoping review

Study selection: A defined and systematic approach was used to collect data. An initial search resulted in 612 articles from PubMed®, MEDLINE®, and EMBASE® databases, with an additional 9 articles added after a manual search. Of these, 548 papers were eliminated in the initial screening phase for various reasons, including language barriers (non-English papers), lack of abstract or full-text availability, non-peer-reviewed status, or being case series or reports. In the subsequent screening, 52 more articles were excluded for reasons such as being in a non-English language, irrelevance to antimicrobial or wound healing studies, not involving PRP or PRF in wound healing, or focusing on PRP use in dental/oral conditions that differ from surgical environments. Ultimately, 12 articles were selected for inclusion in this scoping review and narrative analysis, as mentioned in Figure 1, focusing on the antibacterial effects and wound-healing properties of PRP and PRF.

Data Extraction

An instrument created by the evaluators will be utilized by at least two independent evaluators to extract data from the included publications. This extraction will cover specific details about participants, concepts, context, methodologies, and key findings relevant to the review's questions.

Results

The efficacy of PRP and PRF has been evaluated in various studies, which are summarized as follows (Tables 1-4).

**Table 1:** In-vitro Studies on PRP and PRF PRP: Platelet-Rich Plasma; PRF: Platelet-Rich Fibrin; L-PRP: Leukocyte-PRP; P-PRP: Pure-PRP

Author(s)	Method	Findings	Conclusion	Level of evidence
Bielecki et al. [37]	Kirby-Bauer disc-diffusion method on Mueller-Hinton agar	PRP and PG are active against *S. aureus* and *E. coli*, no activity against other strains	No direct correlation between antimicrobial activity and platelet/leukocyte count	3
Cieslik-Bielecka et al. [38]	Kirby-Bauer disc diffusion method, various thrombin, and calcium chloride activations	Significant correlation between leukocyte subtype and antibacterial effect of L-PRP	L-PRP exhibited leukocyte subtype mediated antibacterial activity against several bacteria	3
Li et al. [39]	Co-culture of HaCAT cells with S. aureus under high glucose condition	EPG and PRG showed significant anti-*S. aureus* activity, reduction in inflammatory response	EPG protects HaCAT cells and promotes proliferation	3
Mariani et al. [11]	Comparison of L-PRP and P-PRP against various bacteria	L-PRP and P-PRP exhibited a time-dependent antibacterial effect	Strong correlation between microcidal protein release and bacterial inhibition	3
Drago et al. [40]	Testing against various strains with different concentrations and activations	Activated P-PRP samples displayed antibacterial activity, dependent on the concentration	Platelet concentration and activation state impact antimicrobial activity	3

**Table 2:** In-vivo studies on PRP PRP: Platelet-Rich Plasma; MRSA: Methicillin-resistant *Staphylococcus aureus*

Author(s)	Method	Findings	Conclusion	Level of evidence
Farghali et al. [41]	Comparison of autologous PRP with topical clindamycin in MRSA-infected wounds on dogs	PRP treatment led to smaller wound size and increased healing rates	PRP, when activated with calcium chloride, had a strong effect on MRSA	3
Yassin et al. [42]	Use of PRP wafers and powder in animal models	PRP wafers and powder showed effective antibacterial and healing properties	PRP wafers may be an effective delivery system for wound application	3

**Table 3:** Clinical studies on PRP PRP: Platelet-Rich Plasma

Author(s)	Method	Findings	Conclusion	Level of evidence
Tran et al. [43]	Activated PRP applied as fibrin gel on non-healing foot ulcers	Complete ulcer closure after about 7 weeks, no adverse events	Activated PRP injection is an effective treatment method	3
Wozniak et al. [44]	Intradermal PRP injection to ulcer margin, microbial analysis	PRP therapy improved healing and increased variety of bacterial flora in some cases	Local application of PRP reduces colony number, increases microbial variety	3

**Table 4:** In-vitro PRF studies PRF: Platelet-Rich Fibrin; PRP: Platelet-Rich Plasma

Author(s)	Method	Findings	Conclusion	Level of evidence
Badade et al. [45]	Evaluation of antibacterial effects against periodontal pathogens	Significant inhibition of *P. gingivalis* and *A. actinomycetemcomitans* by PRP	PRF showed no activity against these pathogens	3
Kour et al. [46]	Well-diffusion method against periodontal pathogens	I‐PRF showed the widest zone of inhibition for *P. gingivalis*, PRP for *A. actinomycetemcomitans*	I‐PRF and PRP have effective antibacterial properties against periodontal pathogens	3
Feng et al. [47]	Comparison of H-PRF and L-PRF against bacteria in relation to immune cell numbers	H-PRF showed better antibacterial activities, correlated with leukocytes and exudate components	Horizontal centrifugation enhances the antibacterial effects of PRF	3

In vitro, examinations reveal diverse PRP and PRF antibacterial impacts against multiple bacterial strains. Research by Bielecki et al. found that both PRP and platelet-rich gel (PG) were effective against S. aureus and E. colibut, not against strains like K. pneumoniae, E. faecalis, and P. aeruginosa [37]. Notably, they observed no direct link between the antimicrobial effect of PRP and the platelet or leukocyte count in the samples. In another study, Cieslik-Bielecka et al. identified a significant relationship between the antibacterial effect of leukocyte-rich PRP (L-PRP) and specific leukocyte subtypes rather than platelet count, demonstrating leukocyte subtype-mediated antibacterial action against pathogens like methicillin-resistant Staphylococcus aureus (MRSA), methicillin-susceptible Staphylococcus aureus (MSSA), E. faecalis, and P. aeruginosa [38].

Li et al. focused on PRP in diabetic foot ulcer treatment, observing that PRP extract (EPG) and PRG significantly reduced the proliferation of S. aureus in co-cultured human keratinocyte cells [39]. This study highlighted EPG's concentration-dependent effect on cell proliferation protection. Mariani et al. compared L-PRP with pure PRP (P-PRP), showing time-dependent antibacterial effects strongly correlated with the release of microcidal proteins and bacterial inhibition [11]. Drago et al. explored how platelet concentration and activation influence PRP's antimicrobial properties, concluding that activated samples displayed effective antibacterial activity, dependent on platelet concentration and activation status [40].

In vivo studies shed light on PRP's clinical applications. Farghali et al. compared autologous PRP with topical clindamycin in MRSA-infected dog wounds, finding that PRP, especially when activated with calcium chloride, led to smaller wounds and increased healing rates [41]. Yassin et al. tested PRP wafers and powder in animal models, noting their effective antibacterial and healing properties, suggesting PRP wafers as a viable wound treatment delivery system [42].

Clinical research supports PRP's effectiveness in treating non-healing wounds. Tran et al. observed complete healing of diabetic foot ulcers with activated PRP, noting no adverse events [43]. Wozniak et al. examined microbial flora changes in venous leg ulcers after PRP injections, reporting significant healing improvements, albeit with diversified bacterial flora in some cases [44].

PRF studies also highlight its antibacterial potential. Badade et al. found PRP, but not PRF, significantly inhibited periodontal pathogens like P. gingivalis and A. actinomycetemcomitans [45]. Kour et al. demonstrated that injectable-PRF (I‐PRF) had a significant antibacterial effect against P. gingivalis, with PRP showing greater efficacy against A. actinomycetemcomitans [46]. Feng et al. compared H-PRF, made by horizontal centrifugation, with traditional L-PRF, discovering enhanced antibacterial activities against S. aureus and E. coli, correlating with immune cell and exudate component presence in H-PRF [47].

Discussion

Over the past 20 years, a lot of research has been done on the regenerative potential of PCs. Only a few findings about their antimicrobial effects in the literature are currently available [45,46]. Autologous platelets have demonstrated efficacy in enhancing tissue regeneration across a range of orthopedic surgical scenarios, encompassing lumbar spinal fusion, tennis elbow, and non-union [48-54]. Bacterial infection is the most serious complication that hinders tissue regeneration and wound healing. Bacteria can penetrate and colonize the wound's underlying tissues even after stringent disinfection. The dynamic interaction between proteolytic enzymes, bacterial exudates that are abundant in toxins, and the persistent inflammatory response has the capability to modify the fundamental cellular mechanisms that are crucial for both the expansion of cells and the process of wound healing [55,56]. In this meticulous examination of scholarly sources, our objective was to scrutinize the subsequent inquiries for research analysis.

Can PRP and PRF Exhibit Antimicrobial Properties?

Most authors agree that platelet preparations, such as PRP, show varying levels of effectiveness against common wound bacteria like MRSA, MSSA, E. coli (extended-spectrum beta-lactamase), K. pneumonia, E. faecalis, P. aeruginosa, B. megaterium, P. mirabilis, E. cloacae, B. cereus, B. subtilis, S. epidermidis, and A. baumannii[12,28,32,57]. Nevertheless, the effectiveness of these preparations varies, especially against specific bacterial species such as P. mirabilis and P. aeruginosa. This variation in efficacy is similar to how specific antibiotics work against different bacterial species, suggesting that PRP might not serve as a uniform treatment option for all bacterial infections. The lack of a standardized method in PRP processing aimed at achieving optimal platelet, leukocyte, cellular component, and antimicrobial protein levels adds complexity and variability. This variability makes it challenging to assess the effectiveness of PRP treatments. The effectiveness of PRP and PRF treatments are evaluated by estimating the amount of growth factors present in the injectate. Additionally, the complexity of the wound environment, often characterized by polymicrobial infections, makes therapy effectiveness dependent on both the wound type and the patient's overall health. This complexity underscores the need for a tailored approach to using PRP for wound infections.

What is the Mechanism by Which PRP Exhibits Antibacterial Activity?

The antimicrobial proteins and peptides of innate immune defense found in platelets, along with the complement and complement-binding proteins present in platelet α-granules, have been suggested as potential contributors to the antimicrobial action of PRP [32,58-62]. It has also been proposed that platelets directly engage with microbes and antibody-dependent cell cytotoxicity, while WBCs directly kill bacteria, produce myeloperoxidase, activate the antioxidant-responsive element, and mount an antigen-specific immune response [32,63]. The function of leucocytes within PCs is highly debated. Several authors have proposed that including white blood cells in PCs may improve scaffold stability and antibacterial capabilities [15]. However, Anitua et al. reported that the higher leucocyte dosage did not significantly increase the antibacterial action of P-PRP [58]. The metalloproteases, pro-inflammatory proteases, and acid hydrolases generated by leukocytes may also intensify the inflammatory reaction at the site of trauma [64].

Is PRP Bactericidal or Bacteriostatic?

PRP exhibits both bactericidal and bacteriostatic properties, meaning it can kill bacteria and inhibit their growth [24,65-68]. The effectiveness of PRP in reaching the minimum inhibitory concentration necessary to halt bacterial replication depends on various factors. These include the bacterial load present in the wound, the overall health condition of the host, the specific type of bacteria involved, and the total quantity of PRP administered. The interplay of these factors determines PRP's efficacy in managing bacterial infections in wounds. In instances where the PRP dosage is insufficient, initial inhibition of bacterial growth may occur, but eventual bacterial overcoming is likely as the antimicrobial effects of PRP diminish over time. Numerous studies suggest that a continuous administration of PRP throughout the wound healing process yields greater benefits compared to a single treatment [41].

Does the Use of Various Agents Provide a Synergistic Effect?

Bielecki et al. have identified a subset of platelet antimicrobial proteins that exhibit characteristics of chemokines and possess inherent antibacterial effects. Moreover, these proteins have the ability to work synergistically with conventional antibiotics while minimizing the risk of bacterial resistance development [37]. The researchers showcased that antibiotics can potentially enhance the antibacterial properties of L-PRP gel [67,69]. The existence of a genuine synergistic effect or merely the presence of multiple bacterial assault pathways remains uncertain. Furthermore, platelets possess angiogenic properties, and the emergence of new blood vessels at the site of the wound could facilitate the distribution of antibiotics while promoting the natural flow of blood, thereby contributing to the healing process.

Implications

Our study reveals that PRP and PRF demonstrate variable efficacy against common wound pathogens, a factor largely dependent on the concentration of platelets and leukocytes. This insight suggests that careful consideration must be given to the composition of PRP and PRF preparations to optimize their antimicrobial effectiveness in various clinical scenarios. Understanding these mechanisms of PRP and PRF will enable clinicians to tailor treatments more effectively to individual patient needs and wound types. Our findings on the bactericidal and bacteriostatic properties of PRP and PRF are particularly relevant in the context of infection control. These properties suggest that PRP and PRF could be valuable tools in managing wound infections, especially in cases where traditional antibiotics are less effective due to resistance issues. The potential synergistic effect of PRP and PRF with antibiotics presents a promising avenue for enhancing the treatment of wound infections. This synergy could potentially reduce the bacterial load more effectively than either treatment alone. However, more research is needed to identify the most effective combinations and treatment protocols.

Regarding real-world application, the need for standardized preparation and PRP and PRF application methods is paramount. This standardization would ensure patients receive the most effective treatment possible, with consistent outcomes across different clinical settings. Our study suggests that integrating PRP and PRF into clinical practice should be approached with a focus on personalized medicine, considering the unique aspects of each wound and patient condition.

Future Directives

Despite the existing knowledge gaps, the scoping review identified specific considerations regarding the clinical application of autologous PRP in wound infections. These considerations include the preparation of PRP. To obtain, separate, and prepare PCs, it is recommended to utilize an FDA-approved Autologous Platelet Separator System. Moreover, using appropriate activation agents, such as Calcium chloride or a combination of Thrombin, is important to standardize and optimize the activation of PRP.

Preparation methods like horizontal centrifugation of PRF can enhance the separation of cellular layers, resulting in decreased cell accumulation. Moreover, Injectable PRF (I-PRF), as the name implies, offers the advantage of being injectable and has the ability to coagulate within minutes after administration. To more comprehensively understand the potential benefits of this therapy in clinical settings, future research should focus on bridging the existing foundational gaps. This includes addressing issues related to standardization, optimizing preparation methods, and understanding the varying effects of PRP and PRF on different types of wounds and bacterial infections. The highlights are mentioned in Table 5.

**Table 5:** Highlights of antimicrobial effects of platelet-rich plasma and platelet-rich fibrin PRP: Platelet-Rich Plasma; PRF: Platelet-Rich Fibrin

Highlights of antimicrobial effects of platelet-rich plasma and platelet-rich fibrin
PRP and PRF effectiveness varies with platelet and leukocyte concentration, emphasizing the need for optimization in wound infection control.
Immune proteins and peptides of platelets contribute to antimicrobial effects, influenced by bacterial load and platelet concentrate concentration.
PRP/PRF may enhance conventional antibiotics, relevant in combating antibiotic resistance; research is needed for effective combinations and protocols.
Standardized preparation methods are essential for consistent outcomes, with consideration for personalized approaches based on patient needs and wound types.
Further research is vital to understand action mechanisms, refine clinical use, optimize preparation methods, investigate effects on various wounds and infections, and standardize clinical applications for effective patient care.

## Conclusions

As explored in this review, the therapeutic applications of PRP and PRF in infection control demonstrate their noteworthy antimicrobial potential. PRP and PRF exhibit variable effectiveness against common wound pathogens, a variability influenced by factors such as platelet concentration, leukocyte content, and activation methods. The antibacterial properties of these concentrates, attributable to the immune proteins and peptides within platelets, require further elucidation to fully understand their mechanisms of action. The observed bactericidal and bacteriostatic effects, modulated by the initial bacterial load, wound environment, and concentrate dosage, highlight the nuanced nature of their application in clinical settings. The potential synergy between these platelet concentrates and antibiotics, suggesting enhanced antibacterial effectiveness, is a promising avenue for future research.

The imperative for standardized preparation and application methods of PRP and PRF in clinical practice emerges as a key takeaway from this review. Establishing these standardized protocols is vital for harnessing their full potential across various wound types and infection scenarios. Although PRP and PRF are promising tools in the medical arsenal for wound management and infection control, the need for additional research is clear. This research should aim not only to unravel the detailed mechanisms of action but also to refine and standardize the clinical use of these concentrates. Such advancements are essential for fully integrating PRP and PRF into routine clinical practice, ensuring their benefits are maximized for patient care in diverse clinical contexts.
