# Correction: Antimicrobial Effects of Platelet-Rich Plasma and Platelet-Rich Fibrin: A Scoping Review

**DOI:** 10.7759/cureus.c337

**Published:** 2025-10-01

**Authors:** Karan CL, Madhan Jeyaraman, Naveen Jeyaraman, Swaminathan Ramasubramanian, Manish Khanna, Sankalp Yadav

**Affiliations:** 1 Orthopaedics, Sanjay Gandhi Institute of Trauma & Orthopaedics, Bengaluru, IND; 2 Orthopaedics, ACS Medical College and Hospital, Dr. MGR Educational and Research Institute, Chennai, IND; 3 Orthopaedics, Government Medical College, Omandurar Government Estate, Chennai, IND; 4 Orthopaedics, Autonomous State Medical College, Ayodhya, IND; 5 Internal Medicine, Shri Madan Lal Khurana Chest Clinic, New Delhi, IND

This article has been corrected to fix the following typos:

- Abstract: "...and 36 articles were selected for inclusion" corrected to "...and 12 articles were selected for inclusion."
- Figure 1: "Identification of studies via other methods" section added to clarify mention in text of nine articles found via manual search.

